# Meat quality of Pulawska breed pigs and image of longissimus lumborum muscle microstructure compared to commercial DanBred and Naima hybrids

**DOI:** 10.5194/aab-63-293-2020

**Published:** 2020-09-01

**Authors:** Anna Kasprzyk, Joanna Bogucka

**Affiliations:** 1 Department of Pig Breeding and Biotechnology, Institute of Animal Breeding and Biodiversity Conservation, University of Life Sciences, 13 Akademicka, 20-950 Lublin, Poland; 2 Department of Animal Physiology, Physiotherapy and Nutrition, Faculty of Animal Breeding and Biology, UTP University of Science and Technology, 28 Mazowiecka, 85-084 Bydgoszcz, Poland

## Abstract

The objective of this article was the evaluation of selected properties of meat quality including the characteristics of longissimus lumborum (LL) muscle microstructure of Pulawska breed pigs and fatteners of the DanBred and Naima hybrids which are used in national meat production. Three genetic groups of fatteners were studied in the experiment: group I – DanBred hybrid; group II – Naima hybrid; and group III – the Pulawska breed. Pig fattening took place under the same environmental conditions from the starting weight of 30 kg ± 2 kg to 103–105 kg. For the analysis of muscle fibre characteristics and meat quality from each group, 30 animals were selected (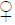
 1:1 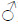). Physico-chemical properties and ultrastructure were evaluated in samples collected from the LL muscle. A statistically significant impact (P<0.01) of a genetic group on pH${}_{45}$, content of water, protein and ash, as well as on the colour of meat, the number of STOs (slow-twitch oxidatives) and the diameter of FTG (fast-twitch glycolytic) muscle fibres, was found. Meat of the Pulawska breed, compared to DanBred and Naima, showed a statistically significant (P<0.01) higher (by 2.05 % and 2.49 %, respectively) nutritional value expressed as protein content and mineral components. Overall, these results imply better biological properties of Pulawska meat than DanBred and Naima hybrids. The higher STO and lower FTG found in muscles from Pulawska pigs might partially explain meat quality differences found between the breeds in the present study. The data of the current study indicated that meat quality characteristics and muscle microstructure of fatteners showed differences, and these differences may be used for alternative pork meat production for the consumer.

## 1 Introduction

Pork meat, when consumed in optimal quantities, is a good source of energy and shows a positive effect on the skin, eyes, nervous and skeletal systems, and mental condition (Singh et al., 2003, cited in Nistor et al., 2012). However, many consumers believe that the quality of raw and processed meat has significantly deteriorated in recent years (Karpiesiuk et al., 2013; Miar et al., 2014). Apart from typical defects such as PSE (pale, soft, exudative), ASE (acid, soft, exudative) and DFD (dark, firm and dry), Tomović et al. (2014) also report on the problem of RSE (red, soft, exudative) and PFN (pale, firm, non-exudative) pork meat. This results, among other factors, from the intensive selection of pig breeds in terms of the accelerated growth of muscle tissue. Intensified synthesis of muscle protein may contribute to physiological changes which have negative consequences with respect to qualitative meat properties (De Vries et al., 1994; Miar et al., 2014). Negative relationships between the quantity and quality of carcass meat are becoming more and more distinct. However, the causes and factors which contribute to this phenomenon still remain unclear.

Given the unlimited market availability, a wide assortment of products and the large supply of meat, the production of high-quality meat which, in the first place, can meet the consumers' expectations has been a very important topic (Nistor et al., 2012; Joo et al., 2013; Miar et al., 2014). In the last decade, the breeding of local breeds which show poorer lean meat percentage and are used for the production of culinary meat and products made in a traditional way has raised greater interest in the United States and some European countries (Hungary, Switzerland, Austria, Germany, Spain, Great Britain and Poland) (Bocian et al., 2012; Nistor et al., 2012)). Raw meat, obtained from autochthonous breeds, shows good quality (Maiorano et al., 2007; Szulc et al., 2012; Kasprzyk et al., 2013). However, the Polish pig meat industry still gives bonuses to producers of fatteners with a high content of meat in the carcass. The introduction of the obligation to classify pork carcasses according to the EUROP system in Poland contributed to the greater interest of national producers in keeping animals of high breeding value. In the country, almost all the new and modernised farms produce fatteners based on imported animals that come from commercial programmes of cross-breeding. Producers choose animals of high breeding value and do not pay attention to the quality of the produced meat. The genotype of the used pigs is a factor which significantly affects meat quality and quantity in commercial breeding focused on the maximisation of profits by achieving a high carcass lean meat content and a fast rate of growth with a small consumption of feed (Ryu et al., 2008; Joo et al., 2013). The available literature provides only information on a few properties of the meat quality of selected breeds but not a comprehensive comparisons with hybrid pigs. Therefore, it is important to gather complete information on the meat quality of animals which are used by the current meat industry because nowadays the good quality of meat should become the main goal in the production of fatteners (Joo et al., 2013).

The objective of the study is the evaluation of selected properties of meat quality, including characteristics of the microstructure of the longissimus lumborum muscle of Pulawska breed pigs and fatteners of the DanBred and Naima hybrids which are used in national meat production.

## 2 Materials and methods

The experiment was conducted according to the recommended EU Directive 2010/63/EU for animal experiments (EU, 2010). Three fattener genetic groups (90 animals) were included in the experiment: group I – DanBred hybrid; group II – Naima hybrid; and group III – the Pulawska breed reared in accordance with welfare requirements. Pigs were fattened from an initial body weight of 30 kg ± 2 kg. The animals were kept in the same environmental conditions. Fattening was divided into two periods. All the pigs were reared under the same diet according to the pigs feeding standards (Grela and Skomiał, 2015). The composition of the complete feed was 13.27 MJ kg${}^{-1}$ of metabolic energy, 167.9 g kg${}^{-1}$ of raw protein and 10.8 g kg${}^{-1}$ of lysine in phase 1 of fattening and 12.61 MJ kg${}^{-1}$ of metabolic energy, 143.8 g kg${}^{-1}$ of raw protein and 8.6 g kg${}^{-1}$ of lysine in phase 2 of fattening. The animals were fed ad libitum with loose feed from feed dispensers and had regular access to water. When the animals obtained 103.0 to 105.0 kg body weight, they were slaughtered at a slaughter house in compliance with the standard commercial procedures of the Polish livestock production system. A total of 90 pigs (30 fatteners from each group – 1:1 gilts to barrows).

At the slaughter line, 45 min after slaughtering, the acidification (pH${}_{1}$) of the longissimus lumborum (LL) muscle was determined in the right half of the carcass behind the last rib. This measurement was performed with a portable digital CPU Star company metre which was equipped with a combined glass electrode. The pH metre was flushed with distilled water after each measurement and re-calibrated every four readings. The pH metre was calibrated before and between measurements with standard phosphate buffers (the pH of the calibrated buffers was 7.00 and 4.00, respectively) (PN-ISO 2917:2001). After a 24-hour cooling down at a temperature of 2–4 ${}^{\circ }$C, the acidity (pH${}_{24}$) of the muscle tissue was determined. In the process of cutting the right half of the carcasses, samples of the LL muscle were collected from the section between the first and third lumbar vertebrae for further chemical and physical testing. The colour was determined on the basis of samples which were 2.5 cm thick and were cut in the plane which was perpendicular to the longitudinal axis of fibres. Meat colour parameters (CIE $L^{*}$, $a^{*}$, $b^{*}$) were determined with an X-Rite Series 8200 spherical spectrophotometer with a hole 12.7 mm in diameter, a D 65 illuminant and a 10${}^{\circ }$ standard observer at 48 h after slaughter. After a 30 min exposure, three colour measurements were made on the freshly cut surface of muscle from each sample. Drip loss was determined at 48 h post-mortem according to the Honikel method (1998). Thus, the slices of meat about 2.5 cm thick which were collected when cutting the carcass were placed in a plastic bag and weighed. Then they were stored at a temperature of +2 ${}^{\circ }$C for 24 h. After that time, the samples were weighed again and drip loss from muscle tissue was calculated based on the difference of weight; this was expressed as a percentage. Thermal drip loss was determined according to the Honikel method (1998). The remaining samples were ground twice in a laboratory grinder with a mesh which consisted of holes 3 mm in diameter, and then they were thoroughly mixed. After the preparation, samples were used to determine the basic chemical composition, water content (drying method according to PN-ISO 1442:2000), protein content (Kjeldahl method according to PN 75/A-04018), fat content (Soxhlet method according to PN-ISO 1444:2000) and mineral compounds in the form of ash (according to PN-ISO 936:2000). Measurements for each sample were performed three times.

Muscle samples 5 mm×15 mm in size were collected from a lumbar section between vertebrae 3 and 4 for the examination of the microstructure 45 min after slaughter, and immediately after collection they were frozen in liquid nitrogen (at a temperature of -196 ${}^{\circ }$C). The frozen meat samples were transferred to a cryostat (Thermo Shandon, United Kingdom) and cut into histological slices which were 10 µm thick at a temperature of about -25 ${}^{\circ }$C. Then the slices were placed on a microscope slide and submitted to an enzyme activity reaction (Ziegan, 1979): NADH-TR tetrazolium reductase (incubation of preparations in the incubation liquid: NADH, NBT, 0.1 M phosphate buffer, pH 7.4, at a temperature of 37 ${}^{\circ }$C for 1 h) and microfibril ATPase (preincubation of preparations in an acidic solution of pH 4.0 for 3 min and then incubation in incubation liquid: ATP, CaCl2, sodium barbiturate, pH 9.6, at 37 ${}^{\circ }$C for 30 min). This reaction enables the determination of the metabolic and contractile type of fibres: STO – red fibres (slow-twitch oxidative; they become dark brown or black after staining); FTO – intermediate (fast-twitch oxidative; they become blue after staining); and FTG – white (fast-twitch glycolytic; they do not stain, light colour). Then microscopic images were saved on a computer disc. A Delta Optical Evolution 300 (Warsaw, Poland) microscope equipped with a ToupCam™ camera was used for that purpose. The percentage content of individual types of muscle fibres was calculated in the MultiScan v. 18.03 (Computer Scanning Systems II Ltd, Warsaw, Poland) programme for computer analysis of the microscopic image, and the diameter of the fibres was measured. The density of muscle fibres was calculated based on the mean number of fibres on a surface area of 1.5 mm${}^{2.}$

The results are expressed as the mean and the standard error (SE) which were analysed using the general linear model procedure of the Statistica AXAP 10.0 software package. Data were subjected to one-way analysis of variance (ANOVA), and a mean comparison was performed (P<0.05, P<0.01) using Tukey procedures.

## 3 Results

In the experiment performed, various indicators of the meat's physical properties were evaluated, and they included acidity, natural and thermal drip loss, and colour (Table 1). When analysing the acidity of the LL muscle, a statistically significant (P<0.01) higher pH${}_{45}$ was found in the Naima and DanBred fatteners compared to fatteners of the Pulawska breed. That parameter was higher by 0.34 of a unit in Naima fatteners compared to fatteners of a local breed. The acidification of the muscle tissue (pH${}_{24}$) in three groups was in the 5.59 and 5.62 range. The reported reduction in pH within 24 h of slaughter shows a normal development of post-slaughter acidification for all the evaluated groups of fatteners.

**Table 1:** Physical properties of the longissimus lumborum muscle in pigs.

Characteristics	Groups		
	DanBred	Naima	Pulawska
pH${}_{45}$	6.46${}^{a}$ ± 0.11	6.53${}^{a}$ ± 0.13	6.19${}^{b}$ ± 0.12
pH${}_{24}$	5.59 ± 0.06	5.62 ± 0.07	5.61 ± 0.14
Drip loss (%)	3.56 ± 1.07	4.63 ± 1.04	3.82 ± 1.15
Thermal drip loss (%)	27.23 ± 1.40	25.45 ± 1.67	26.27 ± 1.75
$L^{*}$	57.80${}^{a}$ ± 0.97	56.59${}^{b}$ ± 1.19	54.94${}^{c}$ ± 1.74
$a^{*}$	0.80${}^{c}$ ± 0.70	1.59${}^{b}$ ± 1.40	1.86${}^{a}$ ± 0.90
$b^{*}$	10.01 ± 0.85	9.93 ± 0.94	9.84 ± 0.46

${}^{a,b,c}$ Values in rows with different letters differ significantly (P<0.01).

In terms of the reported drip loss from the LL muscle which was registered for individual groups of fatteners, no statistical differences were reported. The recorded values of drip loss defined at 48 h post-mortem in the group of DanBred and Pulawska fatteners were at the level of normal meat (without drip loss). No significant differences in thermal drip loss were found between genetic groups of fatteners. When evaluating the parameters of colour, a significant (P<0.01) impact of the group on the brightness of the examined meat samples was found. Raw material from DanBred hybrid fatteners showed the statistically highest value of the $L^{*}$ parameter, and so the meat was bright. The mean value of parameter $L^{*}$ of the meat of the Pulawska breed fatteners was statistically (P<0.01) lower than in the case of DanBred and Naima fatteners. Regarding redness of muscles ($a^{*}$), the Pulawska meat differed (P<0.01) from DanBred and Naima muscles. No statistically significant differences between mean content of a yellow colour ($b^{*}$) was found between the examined muscles of fatteners from individual groups.

**Table 2:** Chemical properties of the longissimus lumborum muscle in pigs (100 g kg${}^{-1}$).

Components	Groups		
	DanBred	Naima	Pulawska
Moisture	75.19${}^{A}$ ± 0.76	75.61${}^{A}$ ± 0.81	73.15${}^{B}$ ± 0.78
Fat	2.50 ± 0.48	2.51 ± 0.57	2.73 ± 0.94
Protein	20.85${}^{A}$ ± 0.68	20.41${}^{A}$ ± 0.98	22.90${}^{B}$ ± 0.38
Ash	0.98${}^{b}$ ± 0.24	0.97${}^{b}$ ± 0.17	1.09${}^{a}$ ± 0.15

${}^{a,\;b}$ Values in rows with different letters differ significantly (P<0.05). ${}^{A,\;B}$ Values in rows with different letters differ significantly (P<0.01).

Table 2 shows data which characterise the content of individual chemical compounds. Statistically significant (P<0.01) differences were found between the content of water and protein in the meat of fatteners of the Pulawska breed compared to the other groups. A higher content of protein and lower content of water were found in the meat of the Pulawska breed fatteners compared to the Naima and DanBred. The level of protein in the muscle of the Naima and DanBred was lower (2.45 % and 2.05 %, respectively) than in the muscle of the Pulawska fatteners. No statistically significant differences between the content of fat were observed in the analysed groups, whereas fat content was higher by 0.23 % in the Pulawska breed fatteners. A statistically significant (P<0.05) higher content of ash was observed in the case of meat of the local Pulawska breed compared to other groups.

**Table 3:** Microstructure of the longissimus lumborum muscle in pigs.

Traits	Fibre	Group		
		DanBred	Naima	Pulawska
Percentage of muscle	STO	9.15${}^{b}$ ± 2.91	9.36${}^{b}$ ± 3.75	17.15${}^{a}$ ± 9.83
fibre (%)	FTO	22.26 ± 5.63	19.78 ± 7.07	19.15 ± 5.97
	FTG	68.59${}^{a,b}$ ± 6.23	70.86${}^{a}$ ± 6.54	63.70${}^{b}$ ± 7.63
Diameter of	STO	45.32 ± 5.15	42.66 ± 6.88	44.28 ± 4.34
muscle fibre (%)	FTO	40.87 ± 3.55	37.49 ± 5.28	35.96 ± 6.65
	FTG	55.01${}^{a}$ ± 3.63	49.30${}^{b}$ ± 5.22	50.00${}^{b}$ ± 5.37
Muscle fibre density	Sum	206.51 ± 30.54	220.57 ± 43.43	233.14 ± 36.96
(fibre number/1.5 mm${}^{2}$)	STO	18.25${}^{B}$ ± 4.23	20.57${}^{B}$ ± 7.60	37.38${}^{A}$ ± 15.31
	FTO	46.13 ± 13.24	43.12 ± 16.32	46.38 ± 18.72
	FTG	142.13 ± 28.42	156.88 ± 36.14	149.38 ± 31.91

${}^{a,\;b}$ Values in rows with different letters differ significantly (P<0.05). ${}^{A,\;B}$ Values in rows with different letters differ significantly (P<0.01). STO stands for slow-twitch oxidative fibres, FTO stands for fast-twitch oxidative fibres and FTG stands for fast-twitch glycolytic fibres. Sum: STO, FTO and FTG fibre number per 1.5 mm${}^{2}$ area of muscle

The analysis of microstructure properties (Table 3) showed that the higher amount of red fibres (STO), which are the most desired fibres from the perspective of meat quality, was found in fatteners of the Pulawska breed (P<0.05). The content of red fibres in muscles of DanBred and Naima breed fatteners, compared to the local breed, was about 8 % lower. There were no significant differences between the examined groups of pigs in the content of intermediate fibres (FTO). The percentage of glycolytic fibres (FTG) in the muscles of the Pulawska fatteners differed (P<0.05) from Naima and DanBred, while the percentage of FTG in the muscles of the Naima did not differ from DanBred (Figs. 2, 3). Statistically significant differences between DanBred breed fatteners and other genotypes were observed in terms of the diameter of FTG muscle fibres. The diameters of glycolytic fibres in muscles of the Naima and the Pulawska breeds were smaller by 5.01 and 5.71 µm, respectively. In addition, higher density of red fibres per examined muscle surface unit was shown in the Pulawska breed pigs (Fig. 3).

**Figure 1:**
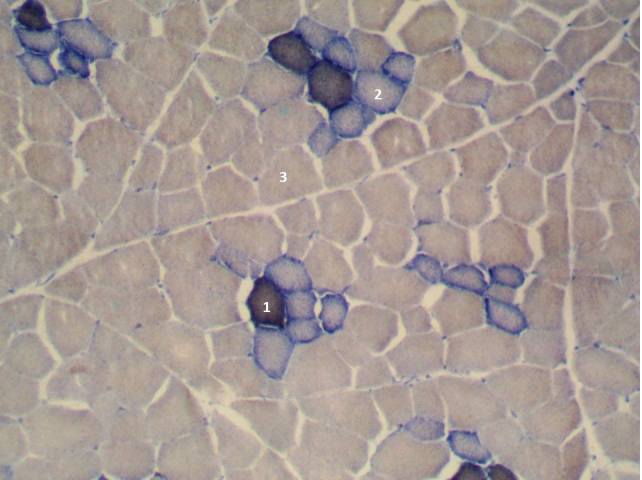
Cross section of the longissimus lumborum muscle in DanBred pig. Magnified 100 times. STO is indicated by 1, FTO is indicated by 2 and FTG is indicated by 3.

**Figure 2:**
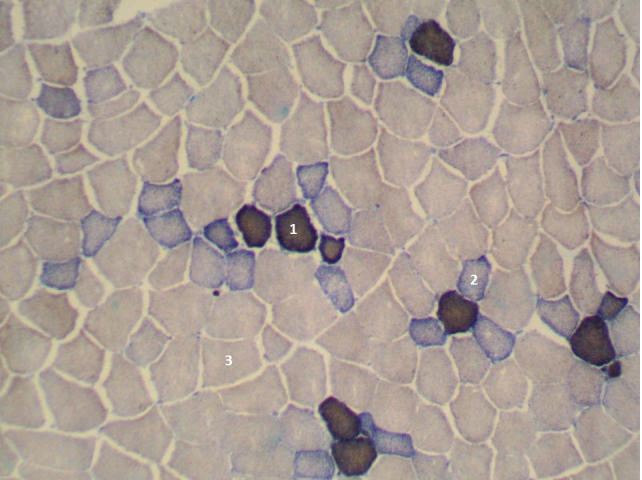
Cross section of the longissimus lumborum muscle in Naima pig. Magnified 100 times. STO is indicated by 1, FTO is indicated by 2 and FTG is indicated by 3.

## 4 Discussion

Technologists from meat production plants find the functional properties of meat very important. This results from the fact that the physico-chemical properties of raw meat have an impact on its storage, preservation and processing, as well as in deciding about the qualitative properties of the finished product. The measurement of pH is used in the meat industry to evaluate meat quality (Kasprzyk et al., 2013). The performance of these tests at two stages, at 45 min and 24 h after slaughter, allows the identification of the most prevalent defects, such as PSE (pale, soft, exudative) and DFD (dark, firm, dry) (Brewer et al., 2001). The acidification of muscle tissue, which is expressed as pH, plays a very important role in the course of glycolytic processes (Choi and Kim, 2009). In addition, it is an essential parameter which needs to be taken into account when evaluating shelf life since acidity is one of the main factors which inhibit the development of bacterial microbiota and thus prevent the spoilage of raw meat. Meat acidity is also affected to a great extent by genetic and environmental factors (Sieczkowska et al., 2009), as well as pre-slaughter (loading and unloading of animals, transport, etc.) proceedings. Raw meat of the present study met the requirements for pork meat of good quality in terms of acidity measured 45 min post-mortem. The results, which were consistent with observations of hybrid fatteners during the authors' study, were reported by Grześkowiak et al. (2009), who analysed the acidity of muscles of Złotnicka Spotted breed fatteners, as well as by Sieczkowska et al. (2009), who measured it in the meat of the Landrace-Yorkshire×Duroc (LY × D) crossbred pigs and in Landrace-Yorkshire×Hampshire (LY × H) breed fatteners (Sieczkowska et al., 2017). Similar acidification of the longissimus muscle (pH${}_{45}$) was found by Babicz et al. (2009) and Piórkowska et al. (2010) in Pulawska breed fatteners and by Szulc et al. (2012) in Złotnicka Spotted breed pigs. The acidification of the muscle tissue at 24 h post-mortem is a very important and significant parameter for meat production plants (Sieczkowska et al., 2009; Kasprzyk et al., 2013). Reported reduction in pH at 24 h post-mortem is an indicator of normal meat maturation. The reported pH${}_{24}$ values for all the genetic groups were typical of normal meat (Stanišić et al., 2016). Values of pH${}_{24}$ of the present study corresponded with the findings reported in the literature (Babicz et al., 2009; Grześkowiak et al., 2009; Piórkowska et al., 2010; Tomović et al., 2016). Wojtysiak and Połtowicz (2014) and Tomović et al. (2016), in accordance with the present study, found ultimate pH values of loin muscles higher in autochthonous breeds than in modern breeds, suggesting that autochthonous breeds could have slower rates of post-mortem pH decline. Sieczkowska et al. (2009) suggested that a higher initial muscle glycogen level of the commercial pig confers an increased capacity for post-mortem glycolysis, or high “glycolytic potential” (GP), that, in turn, enhances the pH decline. Furthermore, the level of glycogen is more affected by genetic than by environmental factors.

**Figure 3:**
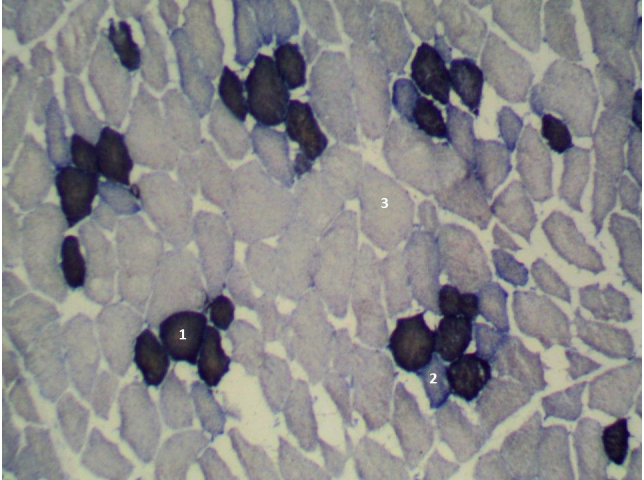
Cross section of the longissimus lumborum muscle in Pulawska pig. Magnified 100 times. STO is indicated by 1, FTO is indicated by 2 and FTG is indicated by 3.

More favourable results in terms of drip loss were reported for the DanBred and the Pulawska breed fatteners. Drip loss from muscles of the Naima breed fatteners was higher by 1.07 % than in the case of the DanBred breed fatteners. The mechanism underlying the retention of water in fresh meat is rooted in the structure of the muscle cell and in the state of key proteins associated with the myofibrils (Huff-Lonergan and Lonergan, 2007). The rate and extent of post-mortem pH decline can affect the quality of meat, especially the drip loss and colour (Bertram et al., 2000; Huff-Lonergan and Lonergan, 2007). The studies of Sieczkowska et al. (2017), which were performed on (Landrace × Yorkshire) × Hampshire breed fatteners showed natural drip loss which was higher by 1.44 % compared to drip loss observed in the meat of the Naima breed fatteners in those studies. In the case of LY × D and Landrace-Yorkshire×Duroc-Pietrain (LY × DP) crossbred pigs, Sieczkowska et al. (2009) reported a natural drip loss of 5.16 % and 7.73 %. Severe natural drip loss from the muscle tissue of pigs during storage is particularly undesired since it is associated with high financial losses (due to loss of meat weight), a reduction in nutritional value, consumer acceptability (limited opportunity to sell it as culinary meat), and the reduced usability and technological performance of meat in the course of its processing (Huff-Lonergan and Lonergan, 2007; Sieczkowska et al., 2009). In the opinion of Joo et al. (1999), high-quality meat, in terms of drip loss at 48 h post-mortem, should show values below 6.0 %. In the case of the stricter classification which is commonly applied in Europe and represented by Bertram et al. (2000), the threshold value of the highest-quality meat (without drip loss) should be at a level of 4.0 %. The intensity of natural drip loss of the muscle tissue, according to Sieczkowska and Iwan (2011), is strictly related to the breed and the variant of crossbreeding. In the authors' opinion, the use of the Duroc breed on the father's side in a commercial crossbreeding (L×Y) × D led to a positive result, i.e. a significant reduction in drip loss from the longissimus lumborum muscle at 48 h after slaughter.

The intensity of thermal drip loss from the LL muscle of fatteners from the analysed group was similar to drip loss which was reported in the literature (Kasprzyk et al., 2013; Matoušek et al., 2016). The increased thermal drip loss from the meat of the DanBred breed fatteners which was observed in the authors' study compared to the meat of fatteners from other groups may be explained by the reduced content of proteins which are directly related to water retention. As Szmańko et al. (2002) and other authors (Sośnicki and Domański, 1983) showed, water retention ability is strictly related to histological meat structure. Poorer water retention is observed in fibres of larger diameter.

Another analysed parameter was the colour of the meat. It is the most important factor in the consumer's assessment, and it is decisive in the freshness and healthiness of the meat (Joo et al., 2013). On the other hand, Brewer et al. (2001) emphasise that instrumental measurement of colour brightness ($L^{*}$) is the best indicator of defective pork meat, as well as PSE and/or DFD. However, Mancinini and Hunt (2005) stated that the intensity of the red colour ($a^{*}$) is a more reliable indicator of meat colour than colour brightness ($L^{*}$). The higher the water content and the smaller the water absorption are, the brighter the meat colour will be; thus this type of meat has a reduced suitability for processing. According to Karpiesiuk et al. (2013), it is related to muscle structure which does not let the light penetrate into deeper meat layers, which results in the intensive reflection of light and thus in the high brightness ($L^{*}$) of colour. Meat of the Pulawska breed fatteners showed a different brightness (compared to the DanBred and Naima breeds) and intensity of red colour (compared to DanBred). The stated differences in the brightness of meat colour should be attributed to the presence of a probably higher content of muscle pigment. The content of myoglobin in muscles containing more red muscle fibres is higher than in white muscle fibres (Kołacz, 2007). The parameters of colour ($L^{*}$ and $a^{*}$) of the LL muscle of the studied Pulawska breed pigs were consistent with the parameters of red meat of other local pig breeds (Matoušek et al., 2016; Stanišić et al., 2016) and LY×D and LY×DP (Sieczkowska et al., 2009) crossbred pigs. It should be emphasised that the colour brightness of the Pulawska breed fatteners was within the range (from 52 to 55) approved for culinary meat of high quality (Przybylski et al., 2008).

Intramuscular fat (IMF) content is related to organoleptic characteristics of pig meat and influences meat and meat product quality, in particular its tenderness, juiciness and palatability (Wojtysiak and Połtowicz, 2014; Tomović et al., 2016). It is widely held that traditional breeds produce a higher IMF content (Bocian et al., 2012; Serra et al., 1998). These observations are consistent with our research, in which a higher IMF content in the longissimus lumborum muscle was found in Pulawska pigs compared to hybrid pigs. These differences in IMF content are caused by the high lipid synthesis capacity of the autochthonous pig breed (Tomović et al., 2016). However, IMF contents higher than 3.5 % are associated with a high risk of meat rejection due to visible fat (Tomović et al., 2016). Moisture content was the lowest in muscles from Pulawska pigs than hybrid fatteners. Our results were in accordance with previous studies (Serra et al., 1998; Tomović et al., 2016; Stanišić et al., 2016; Debrecéni et al., 2018) showing lower contents of moisture in loin muscles from autochthonous breeds than from modern pigs. The protein content of Pulawska muscles was significantly higher than in hybrid fatteners. However, some authors (Serra et al., 1998; Stanišić et al., 2016; Debreceni et al., 2018) established no statistically significant difference in protein content between autochthonous and modern breeds.

Muscle fibres are key components of skeletal muscles. The biochemical properties of muscle fibres determine the properties, type and rate of post-slaughter changes which significantly affect the meat quality (Ryu et al., 2008). Previous studies showed that the composition of muscle fibres is genetically predetermined (Ruusunen and Puolanne, 1997; Ryu et al., 2008; Wojtysiak and Połtowicz, 2014). According to Choi and Kim (2009), a high number of fibres with small and medium diameters contributes to the improvement of meat quality. In the opinion of Damez and Clerjon (2008), the number, diameter and type of muscle fibres, as well as their biochemical, physiological and histological properties, may lead to changes in meat quality.

Many authors indicate the total number of fibres (TNF) as an important muscle growth factor and determinant of meat quality (Lefaucheur, 2010; Lee et al., 2012; Joo et al., 2013; Kim et al., 2013). The analysis of microstructure properties showed that the highest number of fibres was found in the Pulawska breed and the lowest in fatteners of the DanBred hybrid. The number/density of muscle fibres per surface unit is a property which is directly related to the diameter of muscle fibres. Smaller diameters of muscle fibres in the studied groups of fatteners were found in the Naima and the Pulawska breed fatteners compared to DanBred. According to Bogucka and Kapelański (2016), Pulawska breed pigs, compared to Polish Landrace and Złotnicka Spotted, were characterised by the best structure of muscle since they had the highest density of fibres per square millimetre. Smaller fibre diameters are particularly desired since they have a favourable impact on meat quality and are considered an indicator of a delicate meat structure. Larger diameters of type I, IIA and IIB fibres, according to Wojtysiak et al. (2016), are typical of muscles of Piétrain pigs compared to Pulawska fatteners and Polish Large White (PLW).

According to Joo et al. (2013), fresh meat quality is strictly related to the composition of individual types of fibres. Studies by Weiler et al. (1995) provide evidence that intensive selection aimed at increasing muscle weight may change the composition of muscle fibres by increasing the number of glycolytic fibres (type IIB) and the diameter of muscle fibres compared to local breeds. It should be noted that, in our studies, a higher content of STO fibres was reported in the Pulawska breed fatteners. The higher content of STO fibres noted in the native breed is a characteristic feature of wild boar and primitive breeds (Bogucka et al., 2008; Ryu et al., 2008; Wojtysiak and Połtowicz, 2014). Ryu et al. (2008) found that Berkshire local breed pigs have a higher proportion of type I fibres in the longissimus muscle in comparison to Landrace and Yorkshire breed fatteners. Likewise, Serra et al. (1998) showed a higher proportion of red fibres in Iberian pigs compared to the Landrace breed, and Bogucka and Kapelański (2016) showed a higher proportion of these fibres in the Złotnicka Spotted breed and crossbred pigs (PLW × PL) × (DC × P) compared to the Pulawska breed. The influence of breed on muscle microstructure was also compared by Bocian et al. (2012), who state that there is a significantly higher proportion of type I fibres and a lower proportion of type IIB fibres in the longissimus lumborum muscle of Złotnicka Spotted compared to commercial crossbred pigs. According to Wojtysiak and Połtowicz (2014), autochthonous breeds have a higher content of oxidative fibres in muscles than modern breeds. On the other hand, Dai et al. (2009) did not find any statistically significant difference in the thickness and composition of fibres between the Lantang and Landrace breed pigs. Bogucka et al. (2008) showed that a greater number of oxidative fibres was characteristic for the muscles of wild pigs. In the opinion of the authors, the crossing of wild pigs with the Duroc and PL breed pigs reduced the proportion of oxidative fibres and increased the proportion of glycolytic fibres in crossbreeds compared to wild pigs. In our studies, fibres of the glycolytic type of metabolism (FTG) were dominant in the group of Naima and DanBred hybrid fatteners, which may result from intensive selection. Likewise, Wojtysiak et al. (2016) observed the highest content of type IIB fibres in Piétrain (typical meat breed) pigs. The same authors suggest that a higher proportion of glycolytic fibres (IIB) predisposes meat to the development of PSE. The low content of red fibres, observed in our studies in the LL muscles of the DanBred and Naima hybrids, is consistent with the results of observations performed by other authors in a group of commercial fatteners (Bogucka and Kapelański, 2016; Wojtysiak et al., 2016). This effect can results from intensive selection in hybrids pigs.

## 5 Conclusions

A significant impact of the genetic group on the pH${}_{45}$ of the LL muscle was shown. It was found that the physico-chemical properties (pH${}_{45}$, pH${}_{24}$, $L^{*}$) of the meat of Pulawska breed fatteners were very good and even ideal for normal meat. The meat of Pulawska breed pigs, compared to DanBred and Naima breed fatteners, showed a statistically (P≤0.01) lower content of water and a darker colour. The raw meat of the local breed had higher nutritional value than meat of hybrid pigs, which was confirmed by a higher content of protein and mineral components. A dominance of oxidative fibres and a lower content of glycolytic fibres were found in the muscle of Pulawska breed pigs, which had a significant impact on post-slaughter endogenic properties such as pH and meat colour. Based on the performed studies, it can be concluded that knowledge of post-mortem muscle tissue microstructure may contribute to the proper and objective evaluation of meat quality. These data provide valuable information for meat quality differences of Pulawska fatteners and DanBred and Naima hybrids. The obtained information may help breeders to take proper decisions on the selection of animals for the production of high-quality meat.

## Data Availability

The data from this study can be accessed from the authors upon a reasonable request.

## References

[bib1.bib1] Babicz M, Kamyk P, Stasiak A, Pastwa M (2009). Opportunities to use Puławska pigs for heavy fattener production. Ann Anim Sci.

[bib1.bib2] Bertram HC, Petersen JS, Andersen HJ (2000). Relationship between RN${}^{-}$ genotype and drip loss in meat from Danish pigs. Meat Sci.

[bib1.bib3] Bocian M, Wojtysiak D, Jankowiak H, Cebulska A, Kapelański W, Migdał W (2012). Carcass, meat quality and histochemical traits of *m. longissimus lumborum* from Zlotnicka Spotted pigs and commercial pigs. Folia Biol Kraków.

[bib1.bib4] Bogucka J, Kapelański W (2016). Microstructure of *longissimus lumborum *muscle and meat quality of native Polish pig breeds: Złotnicka Spotted and Puławska. Ann Anim Sci.

[bib1.bib5] Bogucka J, Kapelański W, Elminowska-Wenda G, Walasik K, Lewandowska KL (2008). Comparison of microstructural traits of *Musculus longissimus lumborum* in wild boars, domestic pigs and wild boar/domestic pig hybrids. Arch Tierzucht.

[bib1.bib6] Brewer MS, Zhu LG, Bidner B, Meisinger DJ, McKeith FK (2001). Measuring pork color: effects of bloom time, muscle, pH and relationship to instrumental parameters. Meat Sci.

[bib1.bib7] Choi YM, Kim BC (2009). Muscle fiber characteristics, myofibrillar protein isoforms, and meat quality. Livest Prod Sci.

[bib1.bib8] Dai F, Feng D, Cao Q, Ye H, Zhang C, Xia W, Zuo J (2009). Developmental differences in carcass, meat quality and muscle fibre characteristics
between the Landrace and Chinese native pig. S Afr J Anim Sci.

[bib1.bib9] Damez JL, Clerjon S (2008). Meat quality assessment using biophysical methods related to meat structure. Meat Sci.

[bib1.bib10] Debrecéni O, Lípová P, Bučko O, Cebulska A, Kapelánski W (2018). Effect of pig genotypes from Slovak and Polish breeds on meat quality. Arch Anim Breed.

[bib1.bib11] De Vries AG, Van der Wal PG, Long T, Eikelenboom G, Merks JWM (1994). Genetic parameters of pork quality and production traits in Yorkshire populations. Livest Prod Sci.

[bib1.bib12] EU (2010). Directive 2010/63/EU of the European Parliament and of the Council
of 22 September 2010 on the protection of animals used for scientific purposes. Off. J. Eur. Union.

[bib1.bib13] Grela ER, Skomiał J (2015). Pigs Feeding Standards (PFS): Nutritional recommendations and nutritive value of feed for pigs.

[bib1.bib14] Grześkowiak E, Borys A, Borzuta K, Buczyński JT, Lisiak D (2009). Slaughter value, meat quality and backfat fatty acid profile in Złotnicka Spotted fatteners. Anim Sci Pap Rep.

[bib1.bib15] Honikel KO (1998). Reference methods for the assessment of physical characteristics of meat. Meat Sci.

[bib1.bib16] Huff-Lonergan E, Lonergan SM (2007). New frontiers in understanding drip loss in pork: recent insights on the role of post-mortem muscle biochemistry. J Ani Breed Genet.

[bib1.bib17] Joo ST, Kim GD, Hwang YH, Ryu YC (2013). Control of fresh meat quality through manipulation of muscle fiber characteristics. Meat Sci.

[bib1.bib18] Joo ST, Kauffman RG, Lee S, Kim BC, Park GB (1999). The relationship of sarcoplasmic and myofibrillar protein solubility to colour and water-holding capacity in porcine longissimus muscle. Meat Sci.

[bib1.bib19] Karpiesiuk K, Kozera W, Bugnacka D, Falkowski J (2013). Effect of rearing system conditions of fatteners on meat quality and profile of
fatty acids in *m. longissimus dorsi*. Żywność Nauka Technologia Jakość.

[bib1.bib20] Kasprzyk A, Babicz M, Kamyk-Kamieński P, Lechowski J (2013). Slaughter value and meat quality of Pulawska and Polish Landrace breeds fatteners. Ann UMCS.

[bib1.bib21] Kim GD, Jeong JY, Jung EY, Yang HS, Lim HT, Joo ST (2013). The influence of fibre size distribution of type IIB on carcass traits and meat quality. Meat Sci.

[bib1.bib22] Kołacz T (2007). Meat colour. Gosp Mięsna.

[bib1.bib23] Lee SH, Choe JH, Choi YM, Jung KC, Rhee MS, Hong KC, Lee SK, Ryu YC, Kim BC (2012). The influence of pork quality traits and muscle fiber characteristics on the eating quality of pork from various breeds. Meat Sci.

[bib1.bib24] Lefaucheur L (2010). A second look into fiber typing – relation to meat quality. Meat Sci.

[bib1.bib25] Maiorano G, Cavone C, Paolone K, Pilla F, Gambocorta M, Manchisi A (2007). Effects of slaughter weight and sex on carcass traits and meat quality of Casertana pigs reared outdoors. Ital J Anim Sci.

[bib1.bib26] Mancini RA, Hunt MC (2005). Current research in meat color. Meat Sci.

[bib1.bib27] Matoušek V, Kernerová N, Hyšplerová K, Jirotková D, Brzáková M (2016). Carcass traits and meat quality of prestice black-pied pig breed. Asian Austral J Anim Sci.

[bib1.bib28] Miar Y, Plastow GS, Charagu P, Kemp RA, Van Haandel B, Huisman AE, Zhang CY, McKay RM, Bruce HL, Wang Z (2014). Genetic and phenotypic parameters for carcass and meat quality traits in commercial crossbred pigs. J Anim Sci.

[bib1.bib29] Nistor E, Bampidis V, Pentea M, Prundeanu H, Ciolac V (2012). Nutritional quality of pork produced by Mangalitsa breed. Anim Sci Biotechnol.

[bib1.bib30] Piórkowska K, Tyra M, Rogoz M, Ropka-Molik K, Oczkowicz M, Różycki M (2010). Association of the melanocortin – 4 receptor (MC4R) with feed intake, growth, fitness and carcass composition in pigs raised in Poland. Meat Sci.

[bib1.bib31] Przybylski W, Jaworska D, Czarniecka-Skubina E, Kajak-Siemaszko K (2008). Estimating the possibility of isolating high quality culinary met on the basis of fattener meatiness and colour & pH measurements using cluster analysis. Żywność Nauka Technologia Jakość.

[bib1.bib32] Ruusunen M, Puolanne E (1997). Comparison of histochemical properties of different pig breeds. Meat Sci.

[bib1.bib33] Ryu YC, Choi YM, Lee SH, Shin HG, Choe JH, Kim JM, Hong KC, Kim BC (2008). Comparing the histochemical characteristics and meat quality traits of different pig breeds. Meat Sci.

[bib1.bib34] Serra X, Gil F, Perez-Enciso M, Oliver MA, Vazquez JM, Gispert M, Diaz I, Moreno F, Latorre R, Noguera JL (1998). A comparison of carcass, meat quality and histochemical characteristics of Iberian (Guadyerbas line) and Landrace pigs. Livest Prod Sci.

[bib1.bib35] Sieczkowska H, Iwan R (2011). Utilization of the Duroc breed in commercial production of fatteners. Przegl Hod.

[bib1.bib36] Sieczkowska H, Koćwin-Podsiadła M, Krzęcio E, Antosik K, Zybert A (2009). Quality and technological properties of meat from landrace, Yorkshire × Duroc and Landrace-Yorkshire × Duroc-Pietrain fatteners. Pol J Food Nutr Sci.

[bib1.bib37] Sieczkowska H, Zybert A, Krzęcio-Nieczyporuk E, Antosik K, Tarczyński K, Koćwin-Podsiadła M (2017). Culinary and technological suitability of pork obtained from three-way cross fatteners (Landrace × Yorkshire) × Duroc and (Landrace × Yorkshire) × Hampshire. Rocz Nauk PTZ.

[bib1.bib38] Singh PN, Sabate J, Fraser GE (2003). Does low meat consumption increase life expectancy in humans?. Am J Clin Nutr.

[bib1.bib39] Sośnicki A, Domański J (1983). Occurrence of giant fibers in pig meat tissue and exudative of meat. Gosp. Mięsna.

[bib1.bib40] Stanišić N, Parunović N, Stajić S, Petrović M, Radović Č, Živković D, Petričević M (2016). Differences in meat colour between free-range Swallow Belly Mangalitsa and commercially reared Swedish Landrace pigs during 6 days of vacuum storage. Arch Anim Breed.

[bib1.bib41] Szmańko T, Wyskiel S, Gajewczyk P (2002). Relationship between water holding capacity and histological structure of muscle tissue in pigs. Prace Materiały Zoot, Zeszyt Specjalny.

[bib1.bib42] Szulc K, Skrzypczak E, Buczyński JT, Stanisławski D, Jankowska-Mąkosa A, Knecht D (2012). Evaluation of fattening and slaughter performance and determination of meat quality in Zlotnicka Spotted pigs and their crosses with the Duroc breed. Czech J Anim Sci.

[bib1.bib43] Tomović VM, Zlender BA, Jokanović MR, Tomović MS, Šojić SB, Škaljac SB, Tasić TA, Ikonić PM, Šošo MM, Hromiš NM (2014). Technological quality and composition of the *m. semimembranosus* and *m. longissimus dorsi* from Large White and Landrace pigs. Agric Food Sci.

[bib1.bib44] Tomović VM, Šević R, Jokanović M, Branislav Šojić B, Snežana Škaljac S, Tasić T, Ikonić P, Polak ML, Polak T, Demšar L (2016). Quality traits of *longissimus lumborum* muscle from White Mangalica, Duroc × White Mangalica and Large White pigs reared under intensive conditions and slaughtered at 150 kg live weight: a comparative study. Arch Anim Breed.

[bib1.bib45] Weiler U, Appell HJ, Kermser M, Hofäcker S, Claus R (1995). Consequences of selection on muscle composition, A comparative study on *Gracilis* muscle in wild and domestic pigs. Anat Histol Embryol.

[bib1.bib46] Wojtysiak D, Połtowicz K (2014). Carcass quality, physico-chemical parameters, muscle fibre traits and myosin heavy chain composition of *m. longissimus lumborum* from Pulawska and Polish Large White pigs. Meat Sci.

[bib1.bib47] Wojtysiak D, Górska M, Wojciechowska J (2016). Muscle Fibre Characteristics and Physico-Chemical Parameters of *m. semimembranosus* from Pulawska, Polish Large White and Pietrain Pigs. Folia Biolog Kraków.

[bib1.bib48] Ziegan J (1979). Kombinationen enzymhistochemischer Methoden zur Fasertypendifferenzierung und Beurteilung der Skeletmuskulatur. Acta Histochem.

